# A positive fluid balance is an independent prognostic factor in patients with sepsis

**DOI:** 10.1186/s13054-015-0970-1

**Published:** 2015-06-15

**Authors:** Angela Acheampong, Jean-Louis Vincent

**Affiliations:** Department of Intensive Care, Erasme University Hospital, Université Libre de Bruxelles, 808 Route de Lennik, 1070 Brussels, Belgium

## Abstract

**Introduction:**

Intravenous fluid administration is an essential component of sepsis management, but a positive fluid balance has been associated with worse prognosis. We analyzed whether a positive fluid balance and its persistence over time was an independent prognostic factor in septic patients.

**Methods:**

We prospectively studied fluid intake and output for 7 days in 173 consecutive adult patients treated for sepsis in our Department of Intensive Care.

**Results:**

Of the 173 patients, 59 died (34 %). Mean daily fluid intake was higher in non-survivors than in survivors (59 ± 24 ml/kg vs. 48 ± 23 ml/kg, *p* = 0.03), but output volumes were similar. As a result, the daily fluid balance was more than twice as large in the non-survivors as in the survivors (29 ± 22 vs. 13 ± 19 ml/kg, *p* <0.001). Persistence of a positive fluid balance over time was associated with increased mortality. Using a multivariable time-dependent Cox model, a positive fluid balance was independently associated with higher mortality (adjusted hazard ratio 1.014 [1.007–1.022] per ml/kg increase, *p* <0.001).

**Conclusions:**

Persistence of a positive daily fluid balance over time was quite strongly associated with a higher mortality rate in septic patients.

**Electronic supplementary material:**

The online version of this article (doi:10.1186/s13054-015-0970-1) contains supplementary material, which is available to authorized users.

## Introduction

Sepsis, considered today as a dysregulated inflammatory response to an infection [1], is responsible for considerable morbidity and mortality [2]. Sepsis is often associated with a deficit in effective blood volume, resulting from decreased intake, increased external losses, leakage to the interstitial space, and vasodilation. Hence, large amounts of intravenous fluid are often needed to increase cardiac output and improve peripheral blood flow [3]. However, guiding fluid therapy remains a complex issue as cardiac filling pressures are not reliable, signs of fluid responsiveness are not always easy to interpret, and monitoring techniques all have their limitations [4, 5]. Several studies have shown a relationship between positive fluid balance and mortality [5–12], but whether this represents a simple association or a cause-and-effect relationship remains unsettled. To shed some light on this important question, we studied the relationship between changes in fluid balance over time and outcome in a series of septic patients treated in our institution.

## Methods

This prospective observational study was performed in the 35-bed Department of Intensive Care at Erasme University Hospital, Brussels. The study was approved by the ethics committee of Erasme Hospital (reference P2013/108), who waived the need for informed consent due to the observational nature of the study.

All adult patients admitted to the department during 2012 were included if they met the following criteria: (a) age older than 15 years; (b) suspected or proven infection supported by clinical evidence and/or positive bacteriological data, and treated with antibiotics; (c) sepsis-associated organ failure, as defined by a Sequential Organ Failure Assessment (SOFA) subscore of 3 or 4 [13]; (d) duration of intensive care unit (ICU) stay of more than 48 hours. Three patients readmitted for a different sepsis episode were considered as new patients. Septic shock was defined using standard criteria [1].

Patients were treated according to department policy using the Surviving Sepsis Campaign guidelines [3]. Fluid administration was initially guided by a combination of echocardiography, signs of fluid responsiveness in mechanically ventilated patients who were receiving sedative agents, and repeated measurements of cardiac filling [14]. Subsequently, the amount of intravenous fluid given was guided by a number of variables, including arterial pressure, heart rate, cardiac filling pressures and volumes, cardiac output, central venous oxygen saturations and blood lactate levels [3].

Demographic and bacteriologic data were collected from all patients, as were all relevant elements needed to calculate the SOFA score. We also noted the duration of hospital stay before ICU admission, medical or surgical (emergency or elective) reason for admission, origin (home, ambulance, emergency room, hospital ward, other hospital), length of ICU stay, ICU and hospital survival. The use of diuretics or renal replacement therapy (RRT, hemofiltration and/or hemodialysis) was also noted.

Daily fluid intake was calculated as the sum of all intravenous and oral fluids. The daily fluid output was calculated as the sum of the volumes of urine output, ultrafiltration fluid, drain fluid, and estimated gastrointestinal losses (including stools only in the presence of profound diarrhea). Insensitive losses were not taken into account because they are difficult to assess reliably. Daily fluid balance (according to baseline patient weight) was calculated by subtracting the total fluid output from the total intake. Day 1 was defined as the time between ICU admission and the next morning.

### Statistical analysis

Data are given as means with standard deviation or medians and interquartile ranges for continuous variables and as numbers and percentages for categorical variables. The Shapiro-Wilk test was used, and histograms and normal–quantile plots were examined to verify whether there were significant deviations from the normality assumption of continuous variables. Difference testing between groups was performed using Student’s *t* test, Mann-Whitney test, chi-square test or Fisher’s exact test, as appropriate. Repeated measurements were compared using linear mixed models. Time-dependent Cox models were performed to assess the effect of daily fluid balance on survival in the whole population and in the subgroup of patients with septic shock. Daily fluid balance measurements were considered as the time-dependent covariate. The main effect model was built using a backward stepwise elimination technique. The variables considered in the multivariable modeling were selected based on their *p* value in univariate analysis. The threshold considered was 0.1 as a compromise between the number of variables that could be considered in the multivariable analysis and the number of death events in the analyzed population. Colinearity between variables was checked before modeling. The results are presented as crude and adjusted hazard ratios (HR, aHR) with 95 % confidence intervals (95 % CI). Statistical analyses were performed using IBM SPSS 20 statistical program for Windows (IBM Corporation, Armonk, NY, USA). All statistical tests were two-sided and *p* values of less than 0.05 were considered statistically significant.

## Results

During the study period, 225 patients were treated for sepsis in our department, of whom 52 were excluded because they had an ICU stay <48 hours. Accordingly, we studied 173 patients, 114 (66 %) of whom survived the ICU stay (see Additional file 1).

As expected, the non-survivors had a higher SOFA score on admission than the survivors, and were more likely to have a comorbid cancer or an infection involving Aspergillus (Table 1). In patients with shock (*n* = 135), the non-survivors had a longer shock duration (4 ± 2 days vs. 2 ± 2 days, *p* <0.001) than the survivors.

**Table 1:** Demographic and admission characteristics, type of hospitalization, infectious characteristics and length of stay in non-survivors and survivors

	Patients	Non-survivors	Survivors	*p* value
	*n* = 173	*n* = 59	*n* = 114	
Male	117 (68)	35 (59)	82 (72)	0.093
Age (years)	61 ± 16	63 ± 16	60 ± 16	0.185
Weight (kg)	75 ± 20	75 ± 21	75 ± 20	0.897
Septic shock	135 (78)	57 (97)	78 (68)	<0.001
Duration of shock (days)	3 ± 2	4 ± 2	2 ± 2	<0.001
Comorbidities				
Coronary artery disease	26 (15)	10 (17)	16 (14)	0.611
Hypertension	47 (27)	16 (27)	31 (27)	0.992
COPD	25 (15)	8 (14)	17 (15)	0.81
Cirrhosis	19 (11)	10 (17)	9 (8)	0.071
Diabetes	45 (26)	14 (24)	31 (27)	0.622
Cancer	33 (19)	17 (29)	16 (14)	0.019
Immunosuppression	16 (9)	6 (10)	10 (9)	0.764
Variables at ICU admission				
SOFA score	8.2 ± 3.4	9.0 ± 3.3	7.7 ± 3.3	0.023
Cardiovascular subscore	3 [1–3]	3 [1–4]	3 [1–3]	0.008
Renal subscore	1 [0–2]	1 [0–2]	0 [0–2]	0.198
Coagulation subscore	0 [0–2]	0 [0–2]	0 [0–2]	0.728
Lung subscore	2 [1–3]	2 [1–3]	2 [1–3]	0.059
Hepatic subscore	0 [0–2]	0 [0–2]	0 [0–1]	0.365
Neurological subscore	0 [0–2]	1 [0–3]	0 [0–2]	0.099
CRP (mg/l)	170 ± 140	128 ± 111	193 ± 149	0.01
Lactate (mmol/l)	2.9 ± 2.4	3.8 ± 3.4	2.5 ± 1.5	0.148
Origin				0.487
Emergency room	46 (27)	15 (25)	31 (27)	
Ambulance	12 (7)	2 (3)	10 (9)	
Hospital ward	81 (47)	28 (48)	53 (47)	
Other hospital	34 (20)	14 (24)	20 (18)	
Type of admission				0.644
Medical	103 (60)	38 (64)	65 (57)	
Elective surgery	30 (17)	9 (15)	21 (18)	
Emergency surgery	40 (23)	12 (20)	28 (25)	
Source of sepsis				
Lung	65 (38)	31 (53)	34 (30)	0.003
Abdomen	73 (42)	21 (36)	52 (46)	0.206
Urinary tract	14 (8)	3 (5)	11 (10)	0.297
Catheter/blood infection	14 (8)	6 (10)	8 (7)	0.471
Osteoarticular	2 (1)	0 (0)	2 (2)	0.306
Skin	10 (6)	4 (7)	6 (5)	0.685
Other	8 (5)	2 (3)	6 (5)	0.578
Not found	5 (3)	3 (5)	2 (2)	0.215
Microorganisms				
Gram-negative	113 (65)	39 (66)	74 (65)	0.876
*E. coli*	48 (28)	18 (31)	30 (26)	0.559
Pseudomonas	23 (13)	10 (17)	13 (11)	0.308
Other	75 (43)	27 (46)	46 (40)	0.645
Gram-positive	71 (41)	28 (48)	43 (38)	0.217
Staphylococci	27 (16)	12 (20)	15 (13)	0.217
Streptococci	24 (14)	8 (14)	16 (14)	0.932
Other	26 (15)	11 (19)	15 (13)	0.338
Fungi	33 (19)	14 (24)	19 (17)	0.262
Candida	24 (14)	8 (14)	16 (14)	0.932
Aspergillus	9 (5)	6 (10)	3 (3)	0.034
Other	2 (1)	0 (0)	2 (2)	0.306
Virus	4 (2)	3 (5)	1 (1)	0.081
No microorganisms found	27 (16)	9 (15)	18 (16)	0.937
Number of different microorganisms				0.059
0	27 (16)	9 (15)	18 (16)	
1	65 (38)	18 (31)	47 (41)	
2	52 (30)	16 (27)	36 (32)	
≥3	29 (17)	16 (27)	13 (11)	
Duration of ICU stay (days)	6 [4–10]	7 [4–12]	6 [4–8]	0.17
Duration of hospital stay (days)	31 ± 29	18 ± 19	38 ± 31	<0.001
Time between hospitalization and ICU admission (days)	6 ± 13	8 ± 16	6 ± 11	0.084

Values are given as number (percentage), median [25^th^–75^th^ percentile], or mean ± standard deviation (SD)

*COPD* chronic obstructive pulmonary disease, *CRP* C-reactive protein, *SOFA* Sequential Organ Failure Assessment

**Fig. 1:**
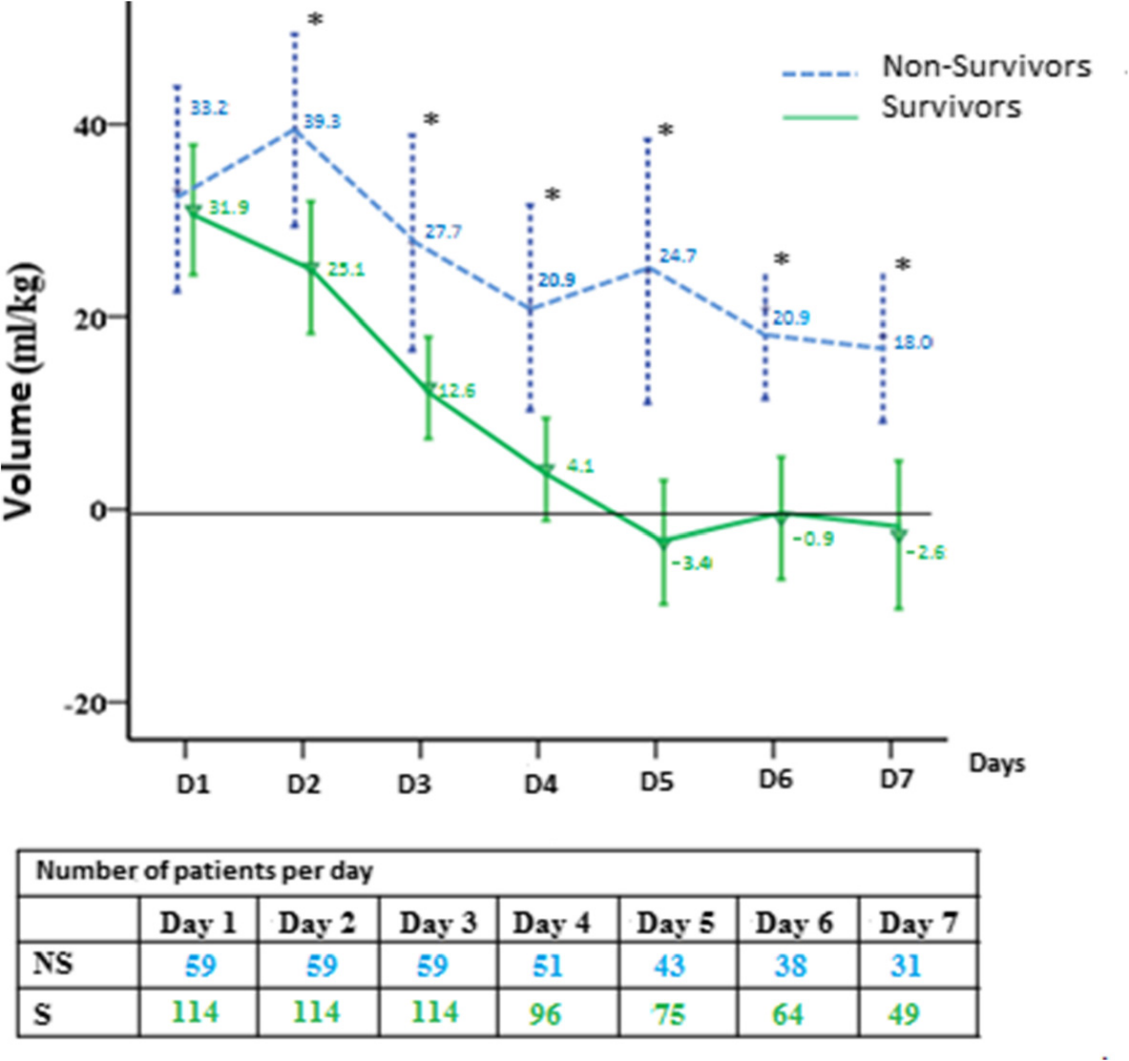
Mean fluid balance (ml/kg) in survivors (S) and non-survivors (NS) over the 7 days after sepsis onset. *Statistically significant difference at the *p* <0.05 level between survivors and non-survivors

**Fig. 2:**
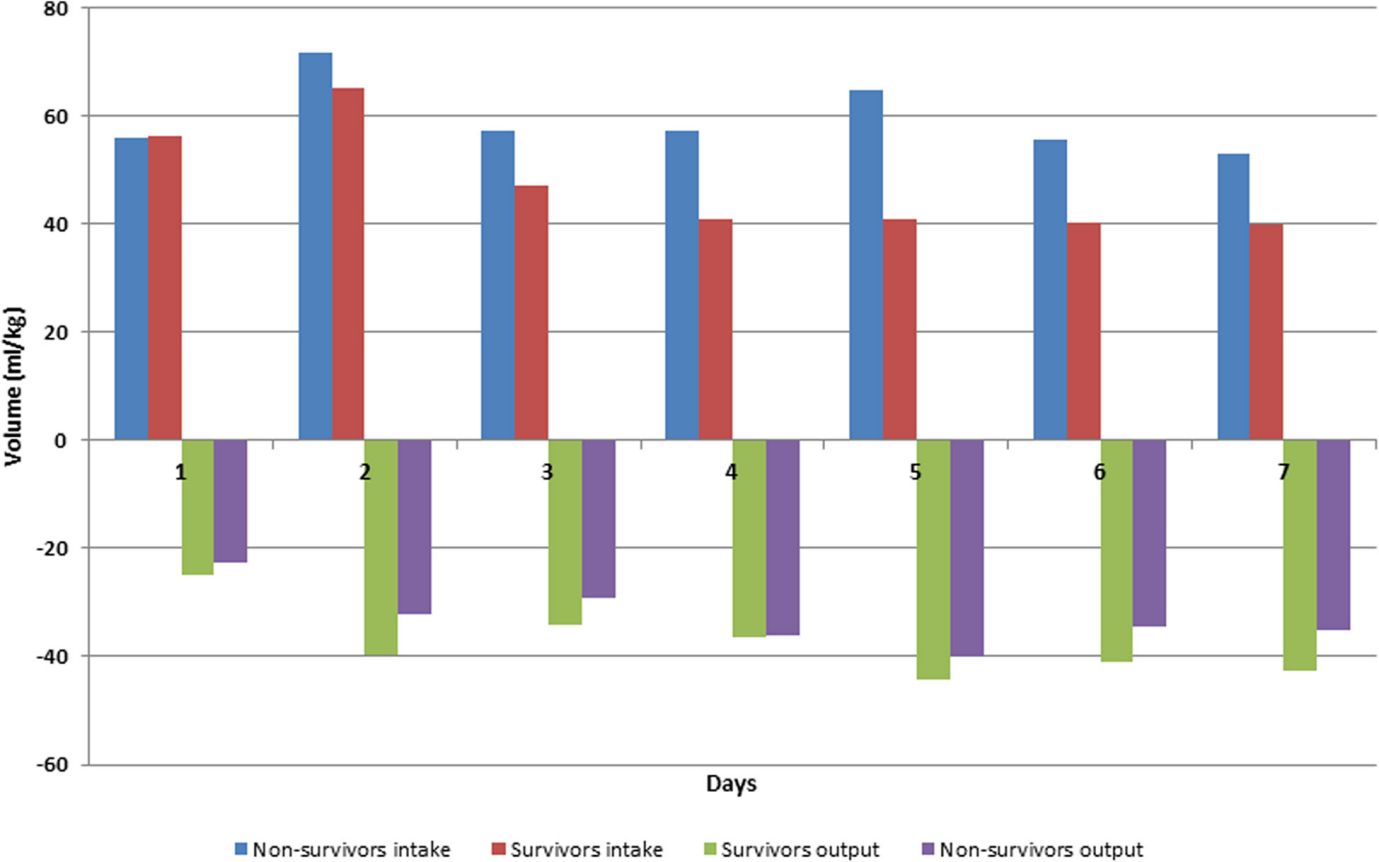
Daily intake and output in non-survivors and survivors during the 7-day period

The total amount of fluid administered for the first three days of ICU stay in all patients averaged 11.8 liters (157 ml/kg. Daily fluid intake was higher in non-survivors than in survivors (*p* = 0.03), but the difference in fluid output was not significant (*p* = 0.49). Overall, the daily fluid balance was more positive in non-survivors than in survivors (*p* <0.01). In the first hours of treatment, the fluid balance was similar in survivors and non-survivors, but from the second day was more positive in non-survivors than in survivors (Fig. 1). Fluid intake decreased and output increased in the survivors while in the non-survivors intake remained higher than output (Fig. 2).

In the survivors, the mean fluid balance became negative between days 4 and 5 and remained negative (Fig. 2). The fluid balance was negative for at least 1 day in 86 of 114 survivors (75 %) but in only 25 of 59 (42 %) of the non-survivors (*p* = 0.01).

**Fig. 3:**
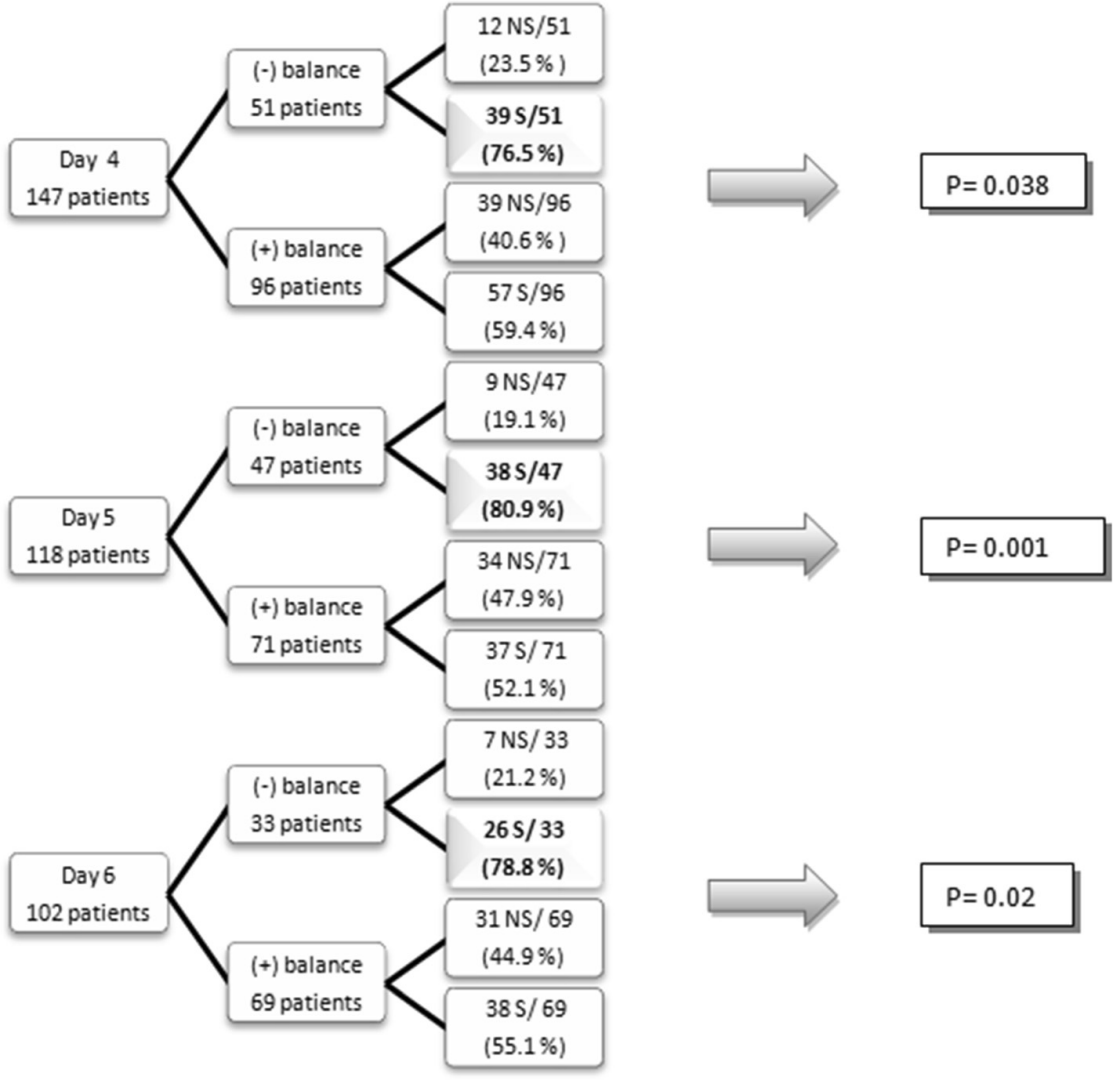
Proportion of patients with a negative fluid balance on days 4, 5 and 6 after ICU admission. On the fourth day, 51 of 147 patients had a negative fluid balance, 39 (76.5 %) of whom were survivors (S). On the fifth day, 47 of 118 patients (39.8 %) had a negative mean fluid balance, 38 (80.9 %) of whom were survivors. And on the sixth day, 33 of 102 patients had a negative fluid balance, 26 (78.8 %) of whom were survivors. *NS* non-survivors

More survivors than non-survivors had a negative fluid balance on the fourth day (76.5 % vs 23.5 %, *p* = 0.038), the fifth day (80.9 % vs 19.1 %, *p* = 0.01) and the sixth day (78.8 % vs 21.2 %, *p* = 0.02) (Fig. 3). Differences in mortality rates according to changes in fluid balance over the first 5 days of the ICU stay are shown in Additional files 2 and 3.

Diuretics were administered in 24 (41 %) non-survivors and 33 (29 %) survivors (*p* = 0.120), while RRT was used in 23 (39 %) non-survivors and 18 (16 %) survivors (*p* = 0.001). We separated patients into those who did not receive diuretics or RRT (“spontaneous diuresis”), those who received diuretics and those who received RRT. In all three groups, non-survivors had a higher daily fluid balance than survivors (Table 2).

**Table 2:** Daily mean fluid intake/output and balance in survivors and non-survivors according to the use of diuretics or renal replacement therapy (RRT)

	Spontaneous diuresis			Use of diuretics			RRT^a^		
	Survivors	Non-survivors	*p* value	Survivors	Non-survivors	*p* value	Survivors	Non-survivors	*p* value
	(*n* = 63)	(*n* = 12)		(*n* = 33)	(*n* = 24)		(*n* = 18)	(*n* = 23)	
Mean daily volume (ml/kg)									
Intake	48 ± 3	55 ± 6	0.31	42 + -3	56 ± 4	0.006	55 ± 8	64 ± 6	0.41
Output	35 ± 2	29 ± 5	0.31	33 ± 2	27 ± 3	0.13	40 ± 5	32 ± 4	0.25
Fluid balance	13 ± 2	26 ± 6	0.03	9 ± 3	28 ± 4	<0.001	16 ± 5	32 ± 6	0.049

Data shown as mean values ± standard error of the mean

^a^Seven patients also received diuretics

Using a time-dependent Cox model in which the daily fluid balance was considered as the time-dependent covariate (see Additional file 4), a positive fluid balance was associated with a higher risk of ICU mortality (HR 1.014 [1.008–1.021] per ml/kg increase, *p* <0.001), a finding which persisted after multivariable modeling (aHR 1.014 [1.007–1.022] per ml/kg increase, *p* <0.001). Other variables that were significantly associated with ICU mortality were age, the type of admission and the presence of cancer. In the subgroup of patients with septic shock, a positive fluid balance was also associated with ICU mortality (aHR 1.013 [1.005–1.020] per ml/kg increase, *p* <0.001).

## Discussion

We studied a single-center, medical-surgical population of 173 septic patients, 78 % of whom had septic shock, with an overall ICU mortality of 34 %. A positive fluid balance was independently associated with an increase in the risk of death. We also observed a relationship between the change in fluid balance over time and mortality.

A positive association between fluid balance and mortality is quite well established. Results from the SOAP study, an observational study of 3,147 adult patients from 198 European ICUs, indicated that, in patients with sepsis, fluid balance was an independent risk factor for mortality [6]. Alsous et al. [7] also showed, in a single-center retrospective study of 36 patients with septic shock, that patients with a negative fluid balance (less than 500 ml) on at least 1 of the first 3 days after the onset of septic shock had better hospital survival. In ICU patients with sepsis or septic shock, Sirvent et al. [10] reported that the accumulated positive fluid balance at 48, 72, and 96 hours was associated with higher mortality, and in a retrospective study, de Oliveira et al. [12] noted that a late (between 24 and 48 hours after diagnosis) positive fluid balance was an independent risk factor for mortality in severe sepsis. In a pediatric septic population, Abulebda et al. [8] showed that a positive fluid balance was associated with worse outcomes (increased mortality and complicated course) in patients with a low initial mortality risk but not in patients at moderate or high risk of death.

However, it is important to consider a time-related relationship, because fluid administration is dynamic, changing according to the patient’s evolution. Recently, it has been suggested that fluid administration for patients in shock should be considered according to the ROSD mnemonic: rescue, optimization, stabilization, and de-escalation phases [15]. We did not focus on the initial, rescue phase of fluid resuscitation, but rather evaluated the time course over several days. Indeed, the role of early goal-directed therapy, including fluid administration, is controversial [16, 17]. In a prospective, multicenter, observational study, Smith and Perner [18] reported that patients with septic shock who initially received a large volume of fluid had improved survival compared to patients who received lower volumes, despite comparable admission severity of illness. However, as noted by Prowle in the accompanying commentary [19], the median of 7.5 liters that was administered in the first 72 hours was a relatively low volume for fluid resuscitation of septic patients. Lee et al. [20] also reported, in a retrospective study, that the initial amount of administered fluid was greater in survivors (at discharge) than in non-survivors. In patients with septic shock complicated by acute respiratory failure, Murphy et al. [21] noted that patients managed with the combination of adequate initial fluid resuscitation and conservative fluid management in the subsequent days had lower in-hospital mortality than other patients.

In our study population, the fluid balance was initially quite similar in the survivors and non-survivors but the non-survivors received more fluids so that already from the second day, the fluid balance was more positive in the non-survivors. After initial resuscitation, less fluid was administered in both groups, and the fluid balance decreased steadily in the survivors but not in the non-survivors. The differences in fluid balance were due to a greater fluid input in the non-survivors rather than to a lower fluid output. Survivors were more likely than non-survivors to have a negative fluid balance early in their ICU stay, and a positive fluid balance was an independent prognostic factor for ICU mortality. The relationship between positive fluid balance and mortality was present regardless of whether or not diuretics or RRT were used.

The single-center nature of our study may be seen as a limitation, but it can also be a strength by limiting variability in patient management as different centers may have different protocols for fluid administration and use of diuretics and RRT. Single-center studies may, therefore, have increased intrinsic validity.

## Conclusions

In critically ill patients with sepsis, a persistent positive fluid balance is quite strongly associated with an increased risk of death. This observation supports the suggestion that fluid administration needs to be carefully titrated after hemodynamic stabilization [15]. Further, interventional studies are needed to confirm these findings.

## Key messages

- Intravenous fluid administration is an essential component of sepsis management
- Accurately determining ongoing fluid requirements in patient with sepsis can be difficult
- Persistence of a positive daily fluid balance over time is quite strongly associated with a higher mortality rate in septic patients

## Additional files

Additional file 1:
**Consort diagram showing flow of patients through the study.**
Additional file 2:
**Mortality rate according to the change in fluid balance over time in patients with a positive fluid balance on day 1.**
Additional file 3:
**Mortality rate according to the change in fluid balance over time in patients with a negative fluid balance on day 1.**
Additional file 4:
**Univariate and multivariable analyses in all patients and the subgroup with shock.**

## Abbreviations

(a)HR: (adjusted) hazard ratio
ICU: intensive care unit
RRT: renal replacement therapy
SOFA: Sequential Organ Failure Assessment
