# Supplementary figures and images for: A positive fluid balance is an independent prognostic factor in patients with sepsis
Source: Crit Care. 2015 Jun 15;19(1):251. doi: 10.1186/s13054-015-0970-1 (PMC4479078; doi:10.1186/s13054-015-0970-1)

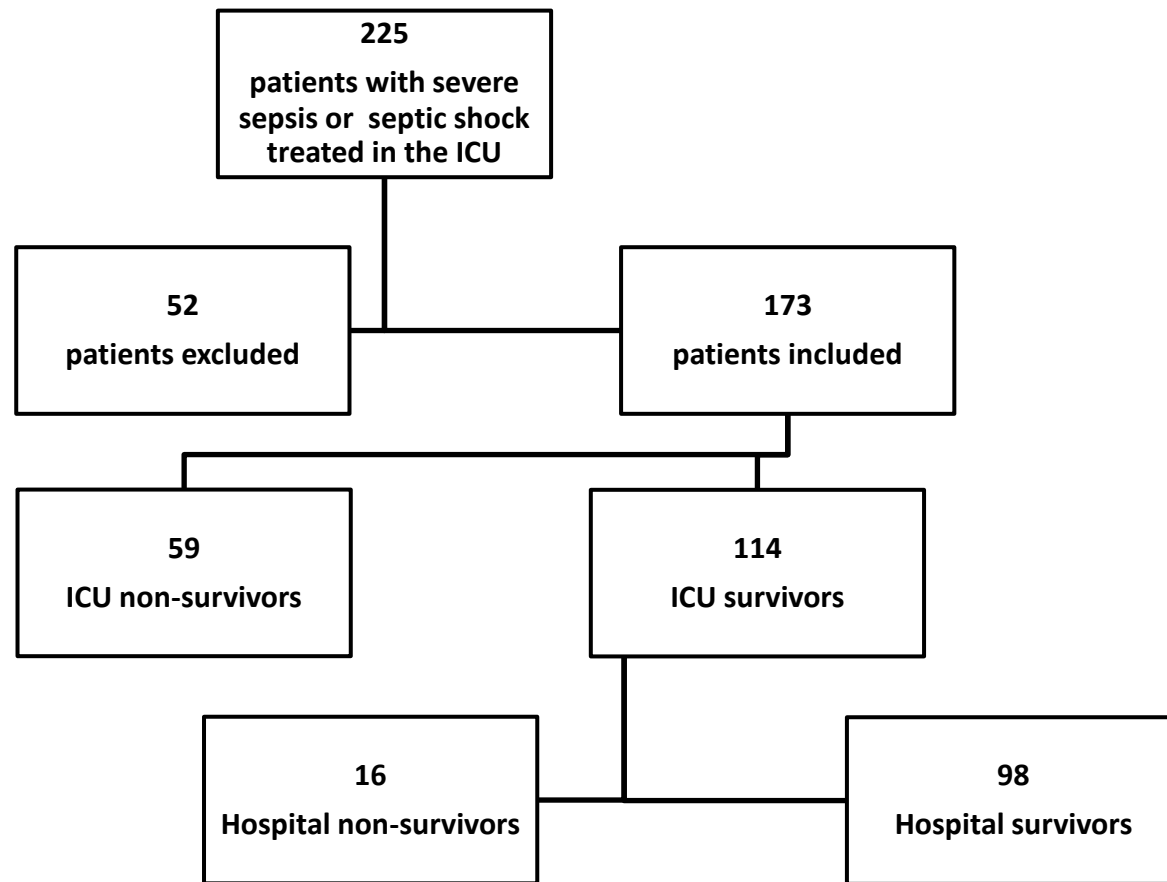

**Additional file 1.** Consort diagram

Supplement: Additional file 1: — Consort diagram showing flow of patients through the study. [file 13054_2015_970_MOESM1_ESM.pdf]
