# Supplementary material for: A positive fluid balance is an independent prognostic factor in patients with sepsis
Source: Crit Care. 2015 Jun 15;19(1):251. doi: 10.1186/s13054-015-0970-1 (PMC4479078; doi:10.1186/s13054-015-0970-1)
Supplement: Additional file 4: — Univariate and multivariable analyses in all patients and the subgroup with shock. [file 13054_2015_970_MOESM4_ESM.pdf]

Additional file 4. Univariate and multivariable analyses in all patients and the subgroup with shock

	All patients				Septic shock patients			
Variables	Univariate		Multivariable		Univariate		Multivariable	
	HR (95% IC)	P-value	aHR (95% IC)	P-value	HR (95% IC)	P-value	aHR (95% IC)	P-value
Age (years)	1.019 (1.003-1.036)	0.02	1.025 (1.005-1.043)	0.01	1.018 (1.002-1.036)	0.03	1.025 (1.005-1.046)	0.01
Male	0.90 (0.53-1.53)	0.7			0.94 (0.55-1.60)	0.81		
SOFA total at admission	1.03 (0.96-1.11)	0.4			1.01 (0.94-1.09)	0.7		
Cardiovascular subscore	1.11 (0.91-1.35)	0.31			0.98 (0.79-1.22)	0.86		
Renal subscore	0.91 (0.71-1.15)	0.43			0.90 (0.70-1.16)	0.42		
Coagulation subscore	1.02 (0.80-1.30)	0.86			1.04 (0.81-1.35)	0.74		
Lung subscore	1.15 (0.93-1.42)	0.19			1.12 (0.91-1.39)	0.29		
Hepatic subscore	1.21 (0.98-1.48)	0.07	1.21 (0.97-1.50)	0.09	1.22 (1.00-1.50)	0.05	1.26 (1.009-1.57)	0.04
Neuro subscore	0.94 (0.80-1.10)	0.42			0.94 (0.80-1.10)	0.43		
Origin								
Emergency room	ref				ref			
Ambulance	0.42 (0.10-1.84)	0.25			0.42 (0.10-1.87)	0.26		
Hospital ward	0.72 (0.38-1.37)	0.31			0.76 (0.40-1.45)	0.41		
Other hospital	0.49 (0.22-1.08)	0.08			0.45 (0.20-1.02)	0.06		
Type of admission								
Medical	ref				ref			
Elective surgery	0.95 (0.40-2.28)	0.91	1.09 (0.43-2.75)	0.86	0.98 (0.41-2.36)	0.97	1.16 (0.45-2.98)	0.76
Emergency surgery	1.72 (0.90-3.31)	0.1	2.29 (1.14-4.58)	0.02	1.78 (0.92-3.42)	0.09	2.40 (1.19-4.83)	0.01
Comorbidities								
Coronary artery disease	1.50 (0.75-3.00)	0.25			1.57 (0.79-3.15)	0.2		
Hypertension	1.19 (0.66-2.13)	0.56			1.21 (0.66-2.22)	0.53		
COPD	1.32 (0.62-2.80)	0.48			1.27 (0.59-2.69)	0.54		
Cirrhosis	2.09 (1.04-4.18)	0.04			2.33 (1.16-4.69)	0.02		
Diabetes	0.82 (0.45-1.50)	0.53			0.92 (0.49-1.71)	0.79		
Cancer	2.65 (1.49-4.70)	<0.001	1.99 (1.09-3.68)	0.03	2.60 (1.46-4.63)	0.001	1.89 (1.03-3.50)	0.04
Immunosuppression	1.60 (0.68-3.79)	0.29			1.46 (0.61-3.45)	0.4		
Diuresis/RRT								
Spontaneous diuresis	ref				ref			

Diuretics	2.05 (1.02-4.11)	0.04			2.04 (1.00-4.18)	0.05		
Renal replacement therapy	1.62 (0.79-3.33)	0.19			1.51 (0.72-3.17)	0.28		
Fluid balance (ml/kg)	1.014 (1.008-1.021)	<0.001	1.014 (1.007-1.022)	<0.001	1.013 (1.006-1.020)	<0.001	1.013 (1.005-1.020)	<0.001
Septic shock	3.86 (0.94-15.91)	0.06			na	na	na	na
