# Supplementary material for: A positive fluid balance is an independent prognostic factor in patients with sepsis
Source: Crit Care. 2015 Jun 15;19(1):251. doi: 10.1186/s13054-015-0970-1 (PMC4479078; doi:10.1186/s13054-015-0970-1)
Supplement: Additional file 2: — Mortality rate according to the change in fluid balance over time in patients with a positive fluid balance on day 1. [file 13054_2015_970_MOESM2_ESM.pdf]

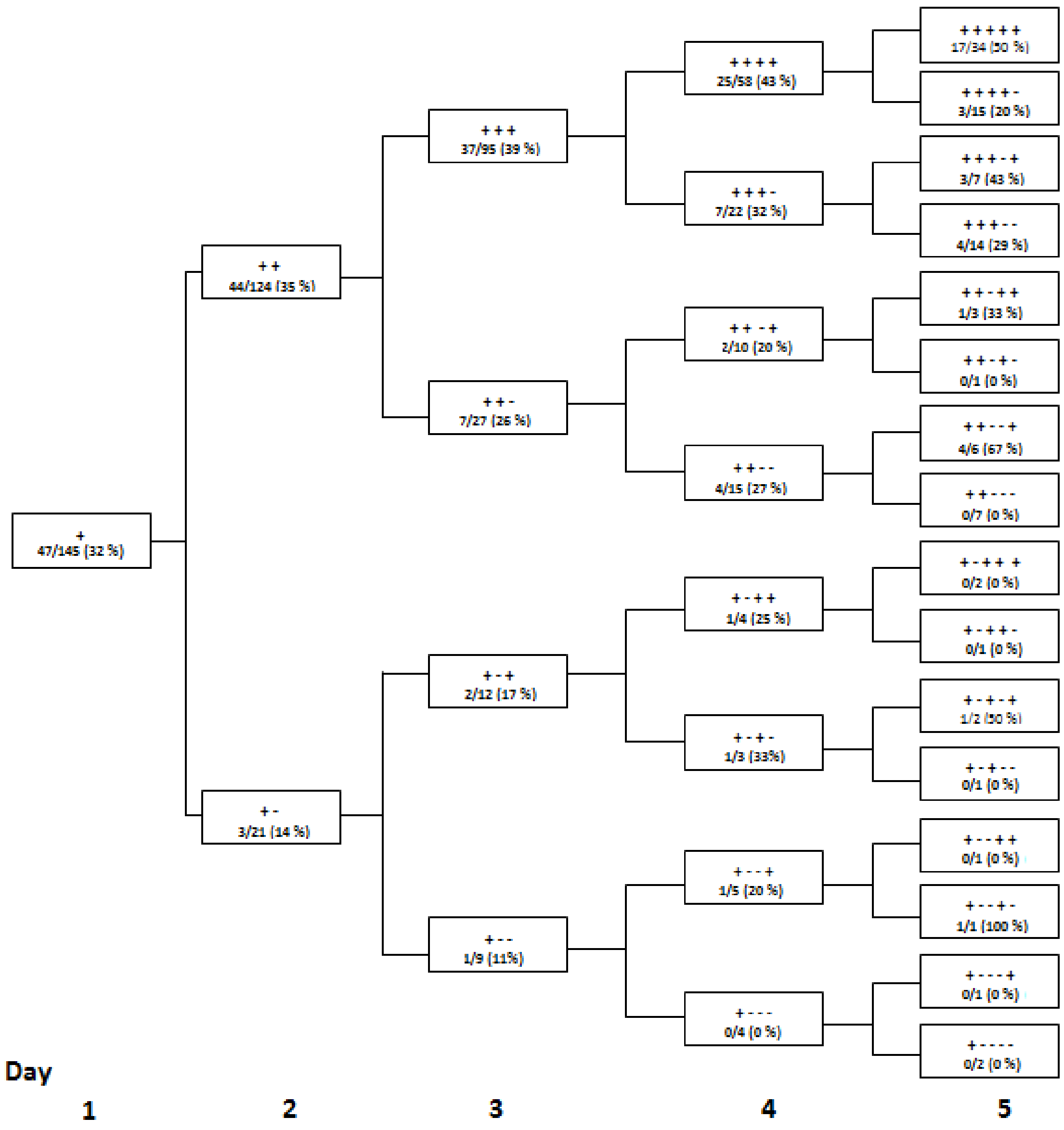

Additional file 2. Mortality rate according to the change in fluid balance in patients with a positive fluid balance on day 1.
